# Public knowledge of dehydration and fluid intake practices: variation by participants’ characteristics

**DOI:** 10.1186/s12889-018-6252-5

**Published:** 2018-12-05

**Authors:** Naila A. Shaheen, Abdulrahman A. Alqahtani, Hussam Assiri, Reem Alkhodair, Mohamed A. Hussein

**Affiliations:** 1 Department of Biostatistics and Bioinformatics, King Abdullah International Medical Research Center (KAIMRC), Ministry of National Guard Health Affairs (MNGHA), Riyadh, Kingdom of Saudi Arabia; 2 King Saud bin Abdulaziz University for Health Sciences, Riyadh, Kingdom of Saudi Arabia; 3 King Khalid University, Abha, Kingdom of Saudi Arabia; 4 Muhammad ibn Saud Islamic University, Riyadh, Kingdom of Saudi Arabia

**Keywords:** Dehydration, Dehydration symptoms, Dehydration causes, Water intake, Fluid intake

## Abstract

**Background:**

Dehydration results from a decrease in total body water content either due to less intake or more fluid loss. Common symptoms of dehydration are dry mouth/tongue, thirst, headache, and lethargy. The aim of this study was to assess knowledge of dehydration definition, symptoms, causes, prevention, water intake recommendations and water intake practices among people living in Riyadh, Saudi Arabia.

**Methods:**

A cross-sectional study using self-reported questionnaire was employed. Participants were invited during their visit to shopping malls. The shopping malls were selected based on geographical location covering east, west, north and southern part of the city. Self-filled questionnaires were distributed to 393 participants, using systematic sampling technique. Variables recorded included demographics, past medical history, knowledge of dehydration definition, symptoms, causes, prevention and daily water intake practices. Descriptive statistics were summarised as mean, standard deviation and proportions. Negative binomial model was used to identify the predictors of water intake. Analyses were performed using SAS version 9.4 (SAS Institute, Cary, NC, USA).

**Results:**

Out of 393 participants, 273(70%) were Saudi, 209(53%) were female, average age was 32.32 ± 8.78 years. Majority 366(93%) had good knowledge of dehydration definition, 332(84%) for dehydration prevention, and 293(74%) of water intake recommendation. Top three recognized dehydration symptoms were: dry lips (87%), thirst (84%), dry tongue (76%) and recognized dehydration causes were: diarrhoea (81%), sweating (68%) and vomiting (62%). The less recognized symptoms were fatigue 176(44.78%), lack of focus 171(43.5%), headache/dizziness 160(40.71%), light headedness 117(29.7%), muscle weakness 98(24.94%), rapid breathing 90(22.9%), and muscle cramps 64(16.28%).The participants had reported an average of 5.39 ± 3.32 water glasses intake per day. The total volume of water intake was significantly different between males *n* = 184 (3.935 ± 2.10 l) and females *n* = 209 (3.461 ± 2.59 l) (*p* = 0.046). The participants’ weight status, intake of juice or tea were the significant predictors of more water intake.

**Conclusion:**

The participants displayed good knowledge of dehydration definition, prevention, and water intake recommendation. The participants also displayed good knowledge of the common symptoms, however knowledge was lacking for uncommon symptoms. Moreover, participants had reported adequate water intake, meeting the daily water intake recommendation of ≥3.7 l for men and ≥ 2.7 l for women.

**Electronic supplementary material:**

The online version of this article (10.1186/s12889-018-6252-5) contains supplementary material, which is available to authorized users.

## Background

Water is a vital component of all living cells and extracellular fluids. Water acts as a solvent, regulates body temperature, aids in food digestion and helps regulate the acid-base balance [1].The balance between water intake or loss and electrolytes is essential for a healthy adult [2]. Water deprivation occurs when the balance between water intake and loss is disrupted and causes a state of dehydration [3, 4]. Dehydration can be defined as decrease in total body water content due to fluid loss, diminished fluid intake, or both [4]. Fluid imbalance as dehydration or overhydration is associated with morbidity and mortality particularly in older adults [5].

In healthy adults with normal Body Mass Index (BMI), water accounts for 60% of body weight [1, 6]. A person can become dehydrated if they lose as little as 3% of their body weight from water depletion [6]. The loss of body mass with no water intake is associated with poor memory and attention [7]. Studies have reported dehydration by 1–2%, impairs cognitive performance and impacts psychomotor and memory skills [8–11]. Fluid deficit of 4% decreases performance, causes headaches, irritability, sleepiness and increases respiratory rate with increase in temperature among children [12]. Dehydration also impairs muscle endurance and decreases muscle strength [13]. The fluid depletion of more than 8% can cause death [14].

Healthcare providers are aware of fluid balance states; dehydration, hypohydration, and euhydration. However, public awareness of dehydration is not widely assessed. The knowledge of dehydration is well documented among athletes [11, 15–18]. According to a survey conducted among dieticians, 76% had knowledge for dehydration definition and 55% had knowledge for water intake recommendation [19]. Few studies have reported knowledge of dehydration in China. The unawareness of minimum water intake was 28.4% among adults, interviewed from four cities across China [20]. Another study conducted in China, have reported good awareness of drinking water among primary and secondary school students 84.5% [21]. The hydration status of the university students’ was assessed in a recent study published in 2017. 46.4% students were dehydrated and 59% had inadequate water intake, but more likely to get dehydrated with coffee intake [22]. National Health and Nutrition survey USA has reported 54% prevalence of dehydration between ages 6–19 years [23].

The common symptoms of mild to moderate dehydration are dry mouth/tongue, thirst, headache, lethargy, fatigue, dry skin, muscle weakness, light-headedness, dizziness and a lack of focus. People with severe dehydration can present with sunken eyes, lack of tears, sunken fontanels (specifically among infants), hypotension, tachycardia and, in the worst-case scenario, unconsciousness [4, 24]. There is an inconsistency reported in dehydration knowledge and reported fluid intake practices by students [21].

Water is required to metabolize food, and a healthy person needs 100 ml of water to metabolize 100 cal [25]. The body’s total water requirement is approximately 4.2 l per day for a healthy 70 kg male [26]. Water requirement depends upon the climate and physical activity level of the person. According to the Institute of Medicine (IOM), water intake of ≥3.7 l daily was considered adequate for men, while ≥2.7 l daily hours water intake for women was considered as adequate [27]. A recent national diet survey in Saudi Arabia had reported diet patterns and beverages consumption, however water consumption is not specified [28]. According to dietary guidelines for Saudis 2012, the recommended water intake is 1.5 litre (6 glasses) daily, with no gender specification [29]. The daily water intake recommendations vary for men, women, pregnant/ lactating women, children, and elderly [30]. Patterns of fluid consumption were assessed among adolescents, only 37% water intake was reported out of total fluid consumption [31].

The risk of developing urinary tract infections, renal stones, dental carries, and constipation increases due to dehydration [32–34]. It is recommended to drink enough fluids to prevent dehydration [29]. High fluid intake is associated with the reduction in kidney stone recurrence [32, 35]. Studies have focused on the different population students, athletes and dieticians while reporting dehydration knowledge and water intake practices [18–21]. The dehydration knowledge estimates provided from published studies might have limited generalizability to different disciplines, climates and cultures. Due to the change in climate and hot weather conditions of Saudi Arabia, this study is geared towards assessing knowledge related to dehydration definition, symptoms, causes, and prevention as well to determine the fluid intake practices among people living in Riyadh, Saudi Arabia.

## Methods

A cross-sectional survey of knowledge assessment of dehydration among people living in Riyadh was conducted during summer 2014 after receiving ethical approval from the institutional review board. The questionnaire was developed to focus on public knowledge of dehydration definition, symptoms, causes, prevention, water intake recommendation and fluid intake practices. The survey included 26 questions, with four domains (i) demographic (*n* = 8), (ii) past medical history (*n* = 2), (iii) knowledge (*n* = 7), and (iv) fluid intake practices (*n* = 9) (Additional file 1: Dehydration questionnaire).

### Defining knowledge

The knowledge of dehydration symptoms, causes and consequences of dehydration were based on ‘yes’ and ‘no’ questions. The knowledge of ‘*dehydration definition’* was assessed by a single question “which of the following statements in your understanding describes dehydration”, (i) I can become dehydrated if I don’t drink enough fluids (e.g. water/ milk/juice/tea), (ii) I can become dehydrated if I don’t eat properly (iii) I can become dehydrated if I don’t sleep properly. The knowledge was defined as good if a participant has selected first or second or all three choices. The knowledge was defined as poor if none was selected or only third choice was selected. The knowledge of ‘*dehydration prevention’* was assessed by a question “In your opinion, which of the following reduces risk of dehydration?” (i) drinking enough fluids (water/milk/juice/tea) (ii) by consuming foods with high water content (iii) in hot climate replenish fluids as priority. All choices are correct answers. The knowledge was defined as good if participant has selected all choices or any two choices as ‘yes’. However, the knowledge was defined as poor if first choice was selected as ‘no’. The knowledge of *‘daily water intake recommendation’* was defined good if selected 2 or 3 litres out of choices (1 to 4 litres). The total water intake was calculated based on the number of water glasses and water bottles used per day. Based on the IMO guidelines, ≥3.7 litres daily water intake for men, while ≥2.7 litres daily water intake for women was considered adequate [27]. The IOM guidelines for water was considered as a cut-off since it has specified the total daily water intake for men and women, compared to Saudi guidelines which is not gender specific. The survey was piloted among medical students and was reviewed by the research staff prior to participants’ enrollment.

### Sample size

This study aimed towards assessing the level of dehydration knowledge. Therefore, formula for prevalence estimation was considered. The sample size was estimated based on the expected prevalence of good knowledge of 50% with 5% precision, 95% confidence interval; the required sample size at the time of statistical analysis is 385.

### Study participants

The malls were selected using *cluster sampling* technique based on different geographical sub-regions of Riyadh city (South, North, West and East) in order to obtain a population sample that was representative of the city and covering all socioeconomic areas. Participants were enrolled in the study during their visit to shopping malls using *systematic random sampling*. The sampling of subjects was spread over seven days where each day was divided into morning interval (9:00 am to 12:00 pm) and evening interval (4:00 pm to 10:00 pm). This has resulted in 14 intervals in which 30 subjects were selected systematically by randomly dropping or including the first subject entering the mall during the specific interval and then sampling every 5th subject until the quota of 30 subjects has been reached. This sampling approach captures the fluctuation in the mall visitors’ characteristics during the hour of the day and the day of the week. The self-administered anonymous questionnaire Arabic version was used for Saudi participants; while English version was used for non-Saudi participants. The completed questionnaires were collected immediately after completion in an envelope to maintain the confidentiality of the responses. In total, 413 participants were approached; 20 (5.0%) participants did not agree to participate. The reasons of their non-participation were not recorded. 393 participants had agreed to participate after obtaining verbal consent for participation.

### Statistical analysis

Demographic characteristics were summarised and reported in terms of mean, standard deviation and proportions. Total water intake was compared across gender by using t-test. Negative binomial model was utilized to determine the predictors of water intake. Number of water glasses intake daily was considered as outcome variable. Predictors used were age, gender, education level, BMI (weight status), history of high blood pressure, history of diabetes mellitus, history of heart disease, history of kidney stones, past history of hospitalization due to dehydration, and coffee/juice/tea/soda intake. BMI was calculated from self-reported height and weight. BMI categories were defined as recommended by Centre for Disease Control and Prevention (CDC) underweight (below18.5 kg/m^2^), normal weight (18.5–24.9 kg/m^2^), overweight (25–29.9 kg/m^2^) and obese (30 and above kg/m^2^) [36], Results are reported as rate ratio, 95% confidence interval and *p*-value. Significance was declared at alpha less than 0.05. Analyses were performed using SAS version 9.4 (SAS Institute, Cary, NC, USA).

## Results

Of the 393 participants, 273(70%) were Saudi, 209(53%) were females, 184(47%) were males and the average age of the sample was 32.3 ± 8.8 years (Table 1). Of the total respondents, 255(65%) had received higher education, and 123(31%) were professionals. The reported chronic health conditions were high blood pressure 54(14%), diabetes mellitus 26(6.7%), history of kidney stones 16(4.0%), and heart disease 5(1.27%) (Table1).

**Table 1:** Participants’ Demographic Characteristics

Demographics	Statistics *n = 393*
Age (mean ± SD)	32.32 ± 8.78
Gender n (%)	
Female	209(53.18)
Male	184(46.82)
BMI (mean ± SD)	20.08 ± 4.96
Education Level (highest) n(%)	
Primary & Secondary	16(4.07)
Diploma	122(31.04)
University	255(64.89)
Monthly Income (Saudi Riyals) n(%)	
< 3000	118(30.03)
≥ 3000–4900	52(13.23)
5000–8999	93(23.66)
≥ 9000- 14,999	79(20.10)
≥ 15,000	51(12.98)
Occupation n(%)	
Professionals	123(31.3)
Clerical Support/ Sales Workers	88(22.4)
Housewife	60(15.3)
Managers	44(11.2)
Students	36(9.2)
Armed forces	23(5.9)
Technicians	18(4.6)
Nationality n(%)	
Saudi	273(69.6)
Non- Saudi	120(30.5)
Reported Chronic Health Conditions *n(%)*	
High blood pressure	54(13.74)
Diabetes mellitus	26(6.62)
Kidney stones	16(4.07)
Heart disease	5(1.27)

The majority of the participants displayed good knowledge of the dehydration definition 366(93%). The knowledge of dehydration prevention 332(84%), and minimum water intake recommendation 293(75%) was good. The knowledge of dehydration consequences was good 312(79%) for kidney stones, while participants had a poor knowledge for death, brain damage and seizures as a dehydration consequences (Table 2).

**Table 2:** Knowledge of dehydration definition, prevention, consequences, and water intake recommendation

Knowledge Questions *n = 393*	Selected	Not Selected
Knowledge of dehydration definition	*n(%)*	*n(%)*
Which of the following statements in your understanding describes dehydration?		
I can become dehydrated if I don’t drink enough fluids (e.g. water/milk/juice/tea)	363(92.37) ^a^	30(7.63)
I can become dehydrated if I don’t eat properly	38(9.67) ^b^	355(90.33)
I can become dehydrated if I don’t get enough sleep	33(8.40) ^c^	360(91.60)
Overall Knowledge	*Good*	*Poor*
	366(93.13)	27(6.87)
Knowledge of dehydration prevention	*n(%)*	*n(%)*
In your opinion, which of the following reduces risk of dehydration?	Correct Choice	Incorrect Choice
Drinking enough fluids (water/milk/juice/tea)	350(89.06)	43(10.94)
By consuming foods with high water content (e.g. watermelon, oranges, apples)	247(62.85)	146(37.15)
In hot climate replenish fluids as priority	350(89.06)	43(10.94)
Overall Knowledge	*Good*	*Poor*
	332(84.48)	61(15.52)
Knowledge of dehydration consequences	*n(%)*	*n(%)*
In your opinion, which of the following conditions may be caused by severe dehydration?	Correct Choice	Incorrect Choice
Kidney stones	312(79.39)	81(20.61)
Death	171(43.51)	222(56.49)
Brain damage	85(21.63)	308(78.73)
Seizure	57(14.50)	336(85.50)
Knowledge of water intake recommendation	*n(%)*	*n(%)*
In your opinion, what is the minimum requirement to drink water for an average weight (70 kg) human?	*Responses*	
1 Litre	-	31(7.89)
2 Litre	150(38.17)	-
3 Litre	143(36.39)	-
4 Litre	-	69(17.56)
Overall Knowledge	*Good*	*Poor*
	293(74.55)	100(25.45)

Selection of ^a^ or ^b^ is correct while ^c^ is incorrect choice

**Fig. 1:**
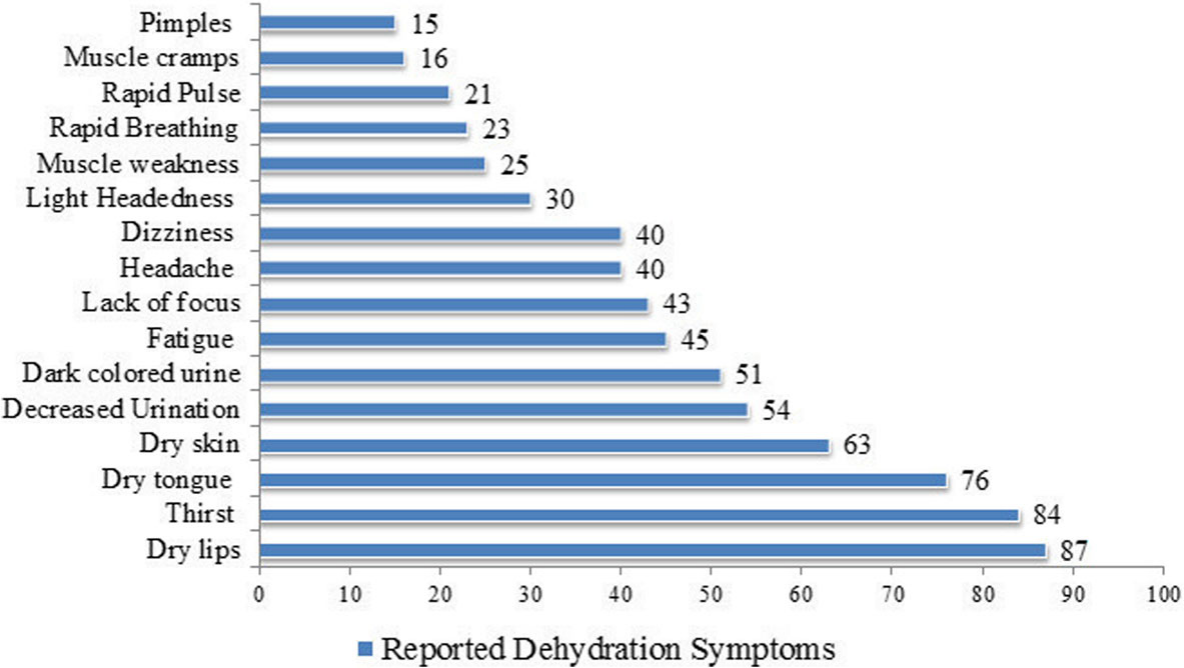
Knowledge of Dehydration Symptoms among Participants

**Fig. 2:**
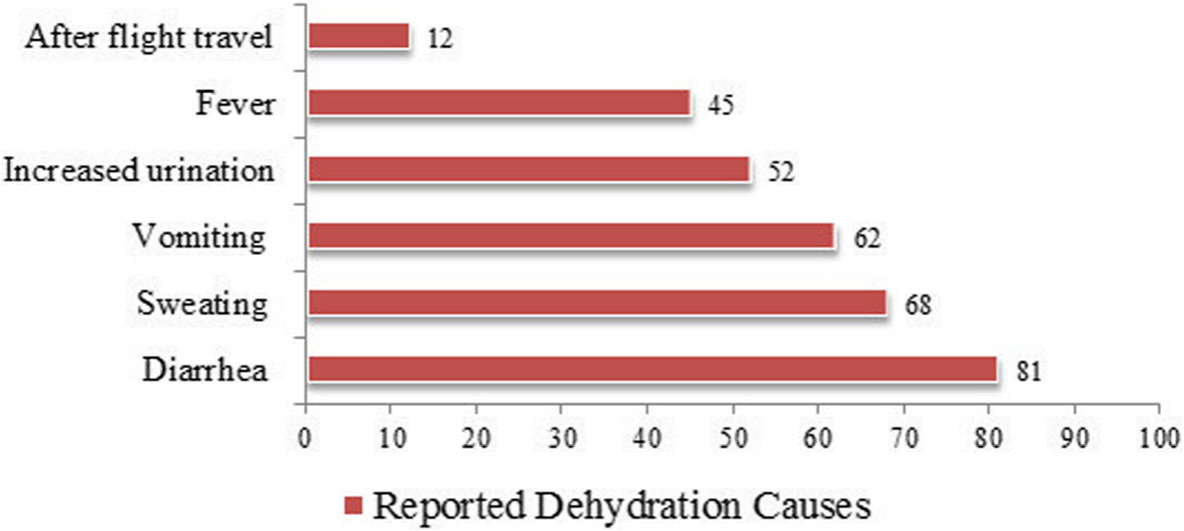
Knowledge of Dehydration Causes among Participants

The most frequently recognized dehydration symptoms were dry lips 341(87%), thirst 329(83.9%), dry tongue 298(75.83%), dry skin 248(63%) and decreased urination 212(53.9%). Moreover, fatigue 176(44.78%), lack of focus 171(43.5%), headache/dizziness 160(40.71%), light headedness 117(29.7%), muscle weakness 98(24.94%), rapid breathing 90(22.9%), and muscle cramps 64(16.28%) were less recognized as dehydration symptoms (Fig. 1). The commonly recognized causes of dehydration were: diarrhoea 319(81%), sweating 264(68%) and vomiting 242(62%), with less recognized causes were increased urination 206(52.42%), and fever 179(45.55%). Only 48(12%) had knowledge that flight travel causes dehydration (Fig. 2).

The self- reported fluid intake by the study participants was summarised in (Additional file 2: Table S1). The participants had reported an average of 5.39 ± 3.32 water glasses intake per day. Only 3(0.75%) participants reported no water consumption, 119(30%) reported drinking 1 to 3 glasses, 188(47.3%) reported drinking 4 to 7 glasses, and 87(22%) reported drinking 8 glasses or more. The total volume of water intake was significantly different between males *n* = 184 (3.935 ± 2.10 l) and females *n* = 209 (3.461 ± 2.59 l) (*p* = 0.046) (Additional file 3: Table S2).

### Predictors of water intake

Less water intake was reported by the participants with increase in age of 10 years (*p* = 0.012), and prior hospitalization due to dehydration (*p* = 0.0003). More water intake was reported by the participants if BMI was underweight (p = < 0.0001) or overweight (*p* = 0.014). The participants were more likely to drink water with intake of additional glasses of juice (*p* = 0.023) and tea (*p* = 0.035) (Table 3).

**Table 3:** Participants’ characteristics and fluid intake across reported water glasses consumption

Predictors	Rate Ratio	95% Confidence Limits	*p*-value*
Intercept	6.782	4.562–10.081	< 0.0001
Age (with 10 years increment)	0.909	0.844–0.980	*0.012*
Gender (males vs *females*)	1.029	0.915–1.158	0.629
Education (diploma vs. *secondary*)	0.786	0.593–1.042	0.095
Education (university vs. *secondary*)	0.870	0.661–1.145	0.322
History of diabetes mellitus (yes vs. *no*)	0.910	0.722–1.147	0.426
History of high blood pressure (yes vs. *no*)	1.150	0.981–1.347	0.083
History of heart disease (yes vs. *no*)	0.957	0.591–1.550	0.859
History of kidney stones (yes vs. *no*)	0.973	0.737–1.284	0.849
BMI (underweight vs. *normal*)	1.724	1.346–2.207	*< 0.0001*
BMI (overweight vs. *normal*)	1.174	1.032–1.336	*0.014*
BMI (obese vs. *normal*)	1.129	0.980–1.301	0.092
Prior hospitalization due to dehydration (yes vs. *no*)	0.772	0.671–0.887	*0.0003*
Coffee intake	1.015	0.984–1.046	0.336
Juice intake	1.056	1.007–1.108	*0.023*
Tea intake	1.038	1.002–1.076	*0.035*
Soda intake	0.980	0.932–1.031	0.445

*p-value is based on the negative binomial model

## Discussion

This study has focused on knowledge of dehydration definition, symptoms, causes, prevention, water intake recommendations and water intake practices conducted at a public level. The published studies assessed dehydration knowledge/or status and water intake practices among students, athletes and dieticians [17–19, 21, 22] .

In this study participants’ displayed good knowledge of dehydration definition. The participants were knowledgeable for the common presenting symptoms of the dehydration; dry lips, thirst, dry tongue, and dry skin. However, knowledge was lacking for the less common symptoms headache, dizziness, light headedness, lack of focus and muscle weakness. Hydration status impacts the perception of dehydration symptoms, as reported in a study the perception of dehydration symptoms (headache, tiredness, poor concentration and thirsty) was different across dehydrated and non-dehydrated students [22].

Despite having the good knowledge of the dehydration definition, the participants had limited knowledge of the causes of dehydration, as well of potentially serious consequences. The knowledge of dehydration consequences, 21% brain damage and 14.5% seizures, was similar to findings from a report of interviews conducted among adults, in which only 14.4% of participants were aware of the harmful effects of dehydration [20]. In a survey conducted among school students in China, 84.5% had knowledge of the consequences of dehydration [21]. By contrast, in this study, more than two-thirds of the participants were lacking knowledge that dehydration can cause brain damage.

Participants had demonstrated good knowledge for dehydration prevention, with the majority reporting that dehydration risk can be reduced by drinking enough fluids. Studies had reported heterogeneous methods of assessing knowledge of adequate water intake based on the target population [19, 20]. In this study 75% participants demonstrated good knowledge for water intake recommendation; higher than 28.4% among people interviewed from four cities in China 28.4% [20].

The water intake practices in this study sample were consistent with IOM water intake recommendation. The total water intake was 3.9 l among men similar to as recommended by IOM (3.7 l daily), and 3.4 l among women higher than the IOM recommendation for women (2.7 l daily) [27]. The water-intake levels reported in this study meets the minimum required daily intake, but do not comply with the 4.1–6.0 l daily intake that is recommended for a healthy, 70 kg adult in an arid climate [30]. The water intake recommendations provided by IOM and Saudi guidelines have some differences. One reason of the differences is that Saudi guidelines have focused on minimum water intake recommendation and did not specify intake based on gender. A healthy adult can be symptomatic even at low levels of dehydration, and the feeling of thirst indicates that one is already dehydrated. To avoid dehydration, fluids should be regularly replenished. Adequate fluid intake is very important when the climate temperature is high and during times of intense physical activity, such as during sports or physical exercise lasting of 30 min or more [3]. The primary and secondary school students in China, have reported good knowledge of dehydration 84.5%, but inconsistent with water intake practices [21]. In contrast, the current study participants had displayed good knowledge of dehydration definition as well reported adequate water intake practices.

Health-care providers, fitness experts and dieticians have raised awareness about the importance of adequate water intake for achieving and maintaining a healthy lifestyle. Food Attitudes and Behaviors Survey, 2007 reported 35% participants with 4 to7 water glasses intake per day, compared to this study sample 47.6%. However intake of eight glasses or more is equally reported 22% [37].

The relationship of several predictors of more water intake was examined in this study. The predictors associated with more water intake were juice, tea intake and weight status. Similarly, the weight status has been reported as a predictor of more water intake in the literature [37]. Age and prior hospitalization were found to be the predictors of less water intake. Similar to the current study, Goodman et al. had reported participants aged 55 years and above drink less compared to younger group [37]. Coffee intake was found to be a predictor of dehydration among university students [22]. In the current study coffee intake and education status was not identified as a compelling predictor of more water intake.

### Limitations

The self-reported water and fluid intake may have been over or underestimated. The sample was recruited from the shopping malls, which imposed selection bias and might be slightly different from the population. For example, in our study we have observed more females (53%) and nationals (70%) compared to population census (40%), (56%) respectively. Our results suggest that water intake differ by gender and therefore our reported overall water intake might not reflect that of the general population. Other limitation of this study is that we did not inquire the activity level and involvement in intense physical activity of the participants, which alters the fluid requirement. The hydration status assessment was beyond the scope of the study.

### Strengths

The study fills a gap in the literature of dehydration knowledge in the Middle Eastern region; given that it’s a hot region with climate change makes hydration essential. The study sample reflects the state of knowledge of general public.

## Conclusion

The participants had displayed good knowledge of dehydration definition, common symptoms and water intake recommendation. Despite having the good knowledge of dehydration definition, the knowledge was lacking for the less common symptoms, causes, and of potentially serious consequences of dehydration. The participants had reported adequate water-intake.

## Additional files

Additional file 1: Dehydration questionnaire Public awareness and knowledge of dehydration in Riyadh, Saudi Arabia. The additional file 1 consists of the dehydration questionnaire. (DOC 201 kb)

Additional file 2: **Table S1.** Reported average fluid intake by the study participants (results table). The additional file 2 consists of reported average water and fluid intake across study participants. (DOCX 14 kb)

Additional file 3: **Table S2.** Reported average fluid intake comparison by gender (results table). The additional file 3 consists of total water intake comparison between males and female. (DOCX 13 kb)

## Abbreviations

BMI: Body mass index
IOM: Institute of Medicine
