# Supplementary material for: Public knowledge of dehydration and fluid intake practices: variation by participants’ characteristics
Source: BMC Public Health. 2018 Dec 5;18:1346. doi: 10.1186/s12889-018-6252-5 (PMC6282244; doi:10.1186/s12889-018-6252-5)
Supplement: Supplementary file 1 — Dehydration questionnaire Public awareness and knowledge of dehydration in Riyadh, Saudi Arabia. The additional file 1 consists of the dehydration questionnaire. (DOC 201 kb) [file 12889_2018_6252_MOESM1_ESM.doc]

**Public Awareness and Knowledge of Dehydration in Riyadh, Saudi Arabia**

Date of Collection:

This is a Research Study to understand people knowledge about dehydration, symptoms of dehydration and preventive measures taken to avoid dehydration.

The answers are confidential and results will be reported in an aggregate form with no reference to subject individual data.

Your participation is completely voluntary.

1. Have you ever participated in questionnaire filling before?

 Yes  No

1. Nationality  Saudi  Non- Saudi
2. What is your age (years): ----------------------------------------
3. What is your height? (Select one scale) -----------cm or ---------- Feet
4. How much is your weight? (Select one scale) ----------kg or ----------pounds
5. Gender:

 Male  Female

1. What is the highest educational level attained? *(Please check only one)*

 Primary

 Secondary

 Diploma

 Higher Education (university)

1. What is your total household income? *(Please check only one)*

 < 3000  ≥ 3000- 4,900  5000 -8,999

 ≥ 9,000- 14,999  ≥ 15,000

1. Have you ever experienced or experiencing any of the below health conditions? *(Select all that applies)*

- Diabetes
- High Blood Pressue
- Heart Disease
- Kidney Stones

1. Which of the following statements in your understanding describes dehydration?

 I can become dehydrated if I don't drink enough fluids (e.g. water/ milk/juice/tea)

 I can become dehydrated if I don’t eat properly.

 I can become dehydrated if I don’t get enough sleep.

1. Which of the following in your opinion could cause body to loose water?

- Sweating  Yes  No
- Fever  Yes  No
- Vomiting  Yes  No
- Diarrhoea  Yes  No
- Increased urination  Yes  No
- After Flight travel  Yes  No

1. In your opinion, restoring fluid loss depends upon below conditions:

- Hot Climate exposure  Yes  No
- Humid climate exposure  Yes  No
- Exercise  Yes  No
- Diarrhoea  Yes  No

13. In your opinion, which of the following could be a symptom of dehydration?

- Thirst  Yes  No
- Dry lips  Yes  No
- Dry tongue  Yes  No
- Headache  Yes  No
- Dizziness  Yes  No
- Dry skin  Yes  No
- Lightheadedness  Yes  No
- Rapid breathing  Yes  No
- Rapid pulse  Yes  No
- Decreased urination  Yes  No
- Dark coloured urine  Yes  No
- Fatigue  Yes  No
- Muscle cramps  Yes  No
- Muscle weakness  Yes  No
- Pimples  Yes  No
- Lack of focus  Yes  No

1. Have you ever been hospitalized due to dehydration?

 Yes  No

15. In your opinion, which of the following conditions may be caused by severe dehydration?

- Kidney stones  Yes  No
- Death  Yes  No
- Brain damage  Yes  No
- Seizure  Yes  No

16. What size of glass you use commonly to drink water: (Select single choice from figures)

17. What size of bottle you use commonly to drink water: *(Select single choice from below figure):*


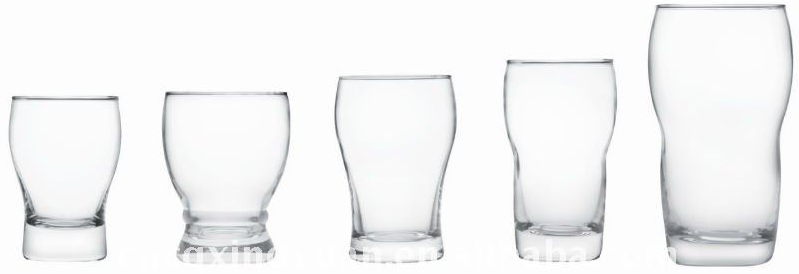


200 mls

300 mls

285 mls

290 mls

655 mls


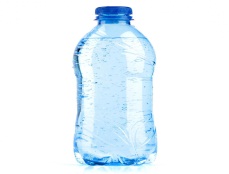


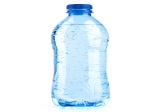


0.6 Liters

0.3 Liters

1. In your opinion, what is the minimum requirement to drink water for an average

weight (70kg) human per day?

 4 Liters  2 Liters  1 Liters  3 Liters

19. How many glasses of water you drink per day during summer?

(Write the number of glasses) ---------------------------------------------

20. How many bottles of water you drink per day during summer?

(Write the number of bottles)--------------------------------------------

1. How many glasses of water have you drunk today?

(Write the number of glasses)--------------------------------------------

1. How many cups of coffee you take usually per day?

(Write the number of cups)--------------------------------------------

1. How many glasses of juice you drink usually per day?

(Write the number of glasses)--------------------------------------------

1. How many cups of tea you drink per day?

(Write the number of cups)--------------------------------------------

1. How many cans of SODA you drink per day?

(Write the number of cans)--------------------------------------------

1. In your opinion, which of the following reduces risk of dehydration?

- Drinking enough fluids (water/milk/juice/tea)  Yes  No
- By consuming foods with high water content (e.g. watermelon,oranges, apple etc )  Yes  No
- In hot climate replenish fluids as priority  Yes  No

*ISCO (international classification of occupations)

27. What is your occupation? (Please select one of below options)

 **Legislators, Senior Officials and** [**Managers**](http://en.wikipedia.org/wiki/Management)

(e.g. chief executives, administrative managers, sales, marketing, information technology, hotel/ restaurant managers, retail)

 [**Professionals**](http://en.wikipedia.org/wiki/Professional)

(e.g. engineers, mathematicians, statisticians, medical doctors, nurses, paramedics, teachers, librarians, lawyers, architects, journalists, [veterinarians](http://en.wikipedia.org/wiki/Veterinarian))

 [**Technicians**](http://en.wikipedia.org/wiki/Technician) **and Associate Professionals**

(e.g. technicians, ship/ aircraft controllers and technicians, medical /[pharmaceutical technicians](http://en.wikipedia.org/wiki/Pharmaceutical_technician), sales agents, fitness workers, telecommunications technicians)

 **Clerical Support Workers**

(E.g. General office clerks, secretaries, customer services, tellers, money collectors)

 **Service and Sales Workers**

(E.g. conductors, cooks, waiters, sales workers, street and market salespersons, shop salespersons, cashiers and ticket clerks)

 **Skilled Agricultural, Forestry and Fishery Workers (farmers**)

 **Craft and related Trades Workers**

(e.g. Building and related trades workers)

 **Plant and Machine Operators and Assemblers**

(e.g. Stationary plant machine operators, chemical/ metal processing plant operators, photographic, rubber, plastic and paper products machine operators, textile, food and related plants, drivers)

 **Elementary Occupations**

(e.g. [Laundry](http://en.wikipedia.org/wiki/Laundry), construction, manufacturing, [manufacturing](http://en.wikipedia.org/wiki/Manufacturing) laborers, transport)

 **Armed forces Occupations**

(e.g. commissioned armed forces officers, non-commissioned armed forces officers, armed forces occupations)

 **Housewife**

 **Student**

**ISCO (international classification of occupations)*
