# Supplementary material for: Public knowledge of dehydration and fluid intake practices: variation by participants’ characteristics
Source: BMC Public Health. 2018 Dec 5;18:1346. doi: 10.1186/s12889-018-6252-5 (PMC6282244; doi:10.1186/s12889-018-6252-5)
Supplement: Supplementary file 2 — Table S1. Reported average fluid intake by the study participants (results table). The additional file 2 consists of reported average water and fluid intake across study participants. (DOCX 14 kb) [file 12889_2018_6252_MOESM2_ESM.docx]

**Supplemental Table 1: Reported average fluid intake by the study participants**

| **Fluid intake questions** *n=393* | *Mean±SD* |
| --- | --- |
| Glasses of water intake per day | 5.39±3.32 |
| Bottles of water intake per day | 3.47±2.44 |
| Cups of coffee intake per day | 1.73±1.75 |
| Cups of juice intake per day | 1.27±1.15 |
| Cups of tea intake per day | 1.41±1.62 |
| Cans of SODA intake per day | 0.85±1.17 |
| **Water Glasses Intake Categories** | *n(%)* |
| *0* | 3(0.77) |
| *1-3 glasses* | 119(29.97) |
| *4-7 glasses* | 188(47.35) |
| *≥ 8 glasses* | 87(21.91) |
