# Supplementary material for: Public knowledge of dehydration and fluid intake practices: variation by participants’ characteristics
Source: BMC Public Health. 2018 Dec 5;18:1346. doi: 10.1186/s12889-018-6252-5 (PMC6282244; doi:10.1186/s12889-018-6252-5)
Supplement: Supplementary file 3 — Table S2. Reported average fluid intake comparison by gender (results table). The additional file 3 consists of total water intake comparison between males and female. (DOCX 13 kb) [file 12889_2018_6252_MOESM3_ESM.docx]

**Supplemental Table 2: Reported average fluid intake comparison by gender**

|  | **Males**  n=184 | **Females**  n=209 | **p-value** |
| --- | --- | --- | --- |
| Total water intake litres (Mean±SD) | 3.935±2.108 | 3.461±2.598 | 0.046* |

**p-value is based on independent sample t-test*
